# Breakdown of hierarchical architecture in cellulose during dilute acid pretreatments

**DOI:** 10.1007/s10570-015-0592-4

**Published:** 2015-02-28

**Authors:** Yan Zhang, Hideyo Inouye, Lin Yang, Michael E. Himmel, Melvin Tucker, Lee Makowski

**Affiliations:** 1 Department of Electrical and Computer Engineering, Northeastern University, Boston, MA 02115 USA; 2 National Synchrotron Light Source, Brookhaven National Laboratory, Upton, NY 11973 USA; 3 Chemical and Biosciences Center, National Renewable Energy Laboratory, Golden, CO 80401 USA; 4 Department of Chemistry and Chemical Biology, Northeastern University, Boston, MA 02115 USA; 5 Department of Bioengineering, Northeastern University, Boston, MA 02115 USA

**Keywords:** Cellulose, Hierarchical architecture, Guinier analysis, Multi-Angle X-ray scattering

## Abstract

Cellulose is an attractive candidate as a feedstock for sustainable bioenergy because of its global abundance. Pretreatment of biomass has significant influence on the chemical availability of cellulose locked in recalcitrant microfibrils. Optimizing pretreatment depends on an understanding of its impact on the microscale and nanoscale molecular architecture. X-ray scattering experiments have been performed on native and pre-treated maize stover and models of cellulose architecture have been derived from these data. Ultra small-angle, very small-angle and small-angle X-ray scattering (USAXS, VSAXS and SAXS) probe three different levels of architectural scale. USAXS and SAXS have been used to study cellulose at two distinct length scales, modeling the fibrils as ~30 Å diameter rods packed into ~0.14 μm diameter bundles. VSAXS is sensitive to structural features at length scales between these two extremes. Detailed analysis of diffraction patterns from untreated and pretreated maize using cylindrical Guinier plots and the derivatives of these plots reveals the presence of substructures within the ~0.14 μm diameter bundles that correspond to grouping of cellulose approximately 30 nm in diameter. These sub-structures are resilient to dilute acid pretreatments but are sensitive to pretreatment when iron sulfate is added. These results provide evidence of the hierarchical arrangement of cellulose at three length scales and the evolution of these arrangements during pre-treatments.

## Introduction

Lignocellulosic biomass is one of the most abundant and low-cost bioenergy resources in the world. The utilization of lignocellulose in maize has huge potential to become a renewable energy supply producing ethanol as well as other fuels. However the limitation of cellulose deconstruction remains a substantial obstacle (Himmel et al. 2007). Chemical pretreatment such as dilute sulfuric acid and ferrous sulfate treated at high temperature can hydrolyze hemicellulose (Nguyen and Tucker 2002; Tucker et al. 2003), transport lignin (Donohoe et al. 2008) and break cellulose chains into fragments (Saeman 1945; Mok and Antal 1992; Inouye et al. 2014).

Cellulose crystal structure has been determined (Nishiyama et al. 2002, 2003) and I alpha and I beta crystal forms have been found in algae and plants (Atalla and VanderHart 1984). Aggregates of cellulose microfibrils have been studied (Xu et al. 2007) and 36- (Endler and Persson 2011; Ding et al. 2012) and 24-chain models (Fernandes et al. 2011; Thomas et al. 2013) have been proposed for the microfibrils. Furthermore, structural changes due to chemical treatments have been characterized (Pingali et al. 2010; Driemeier et al. 2011; Inouye et al. 2014).

X-ray scattering has been used to study the structure of cellulose in very small-angle and wide-angle regimes, corresponding to length scales spanning from 2.2 to 5000 Å. Previous studies (Nishiyama et al. 2014) demonstrated an increase in the apparent crystallite size of cellulose in aspen wood under pretreatment by steam, dilute acid and AFEX-III. Penttilä et al. (2013)showed that hot water pretreatment increases the lateral width of cellulose crystallites and opens pores between bundles of microfibrils. We have shown that acid and acid plus iron pretreatment has little impact on the size of fibril bundles in the 100 nm length scale as judged by USAXS and scanning electron microscopy (SEM) of corn stover (Inouye et al. 2014). However the length scale between 100 and 1000 Å has received less scrutiny as it is difficult to study using either USAXS or SEM. Here, we describe experiments in which we collected X-ray scattering from maize at intermediate-angles in order to bridge the resolution gap and investigate the possibility of structural features intermediate in length scale. The VSAXS data were combined with USAXS and SAXS data to provide multi-scale information about the structural organization of cellulosic architecture in maize.

Guinier analysis in cylindrical coordinates (Iampietro et al. 1998) was applied to USAXS, VSAXS and SAXS data to investigate the structural organization with sizes from 30 to 1000 Å. As in the case of solution scattering, the cylindrical homolog of the Guinier plot, relevant to fiber diffraction, is linear at small angles where it provides information about the radius of gyration (moment of inertia) of the dominant species in the scattering volume. In the case of hierarchical structures, it is predicted that there will be multiple linear regions in the Guinier plot, corresponding to different length scales in the structure. In order to identify these regions, we have used the second derivative of the Guinier plot, which should be zero or near zero when the Guinier plot is linear. A noise suppressing algorithm using total variation regularization (Chartrand 2011) was applied to make possible accurate calculation of the derivatives of the data in the presence of substantial noise.

## Materials and methods

### Pretreated maize samples

Samples used in X-ray scattering experiments were milled and dried maize stover as previously described (Tucker et al. 2003). Corn stover was collected from a single-field from Gustafson Farm, Weld County, CO and the corn stems were prepared from Round-up Ready Pioneer hybrid 36N18. Stalks were milled to 20-mesh in a Thomas Wiley cutting mill and freeze-dried after they were air dried to a moisture content of 10 %. The samples contain both primary and secondary cell walls and samples studied included: untreated, 0.5 % H_2_SO_4_ treated; and 2 mM Fe_2_(SO_4_)_3_ treated samples. The treated samples were heated for 15 min at 150° C. Maize samples used for X-ray scattering experiment were 1–2 mm in length and 0.2–0.5 mm across. The details of mechanical disruption of dilute acid treated corn stover by three types of reactor are shown by Ciesielski et al. (2014) and Wang et al. (2014).

### X-ray scattering experiments

USAXS, VSAXS data were collected at beamline X9 at NSLS at Brookhaven National Laboratory (Allaire and Yang 2010). USAXS data were collected utilizing a sample to detector distance of 4980 mm and X-ray wavelength of 1.55 Å for 10 s exposure time. VSAXS data was collected using a sample to detector distance of 1936 mm and X-ray wavelength of 0.83 Å. SAXS data were collected at GM/CA, beamline 23ID-B at APS at Argonne National Laboratory (Xu et al. 2011) utilizing a sample to detector distance of 300 mm and X-ray wavelength of 1.033 Å.

### Scattering intensity model and Guinier analysis in cylindrical coordinates

Guinier analysis has often been used to analyze scattering intensity at low angles to estimate the radius of gyration (R_g_) of the scattering object in solution scattering (Glatter 1977). An analogous treatment of the equator of fiber patterns can be applied using cylindrical coordinates (Iampietro et al. 1998). Here a cylindrical Guinier analysis model has been utilized and applied to both computationally generated and experimental data to estimate the radius of gyration of substructures within the biomass. Computationally generated intensity distributions for populations of solid cylinders were generated using (Inouye et al. 1993):

1 $$I(q) = \int\limits_{0}^{{r_{m} }} {\left[ {2\pi r\frac{{J_{1} (qr)}}{q}} \right]^{2} \frac{1}{{\sqrt {2\pi } \sigma_{r} }}\exp \left[ { - \frac{{(r_{0} - r)^{2} }}{{2\sigma_{r}^{2} }}} \right]dr} .$$

where I(q) is the equatorial intensity, r_0_ and σ are average radius and standard deviation of a Gaussian population distribution of cylinders, J_1_ is a Bessel function of the first kind, q is the scattering vector in reciprocal space.

Iampietro et al. (1998) derived a plot for fiber diffraction data that is analogous to the Guinier plot in solution scattering. In particular, ln [qI(q)] is a linear function of q^2^ with slope of $- \frac{{R_{xc}^{2} }}{2}$ (Putnam et al. 2007):

2 $$\ln [qI(q)] = \ln [qI(0)] - \frac{{q^{2} R_{xc}^{2} }}{2}$$

where R_xc_ is the moment of inertia of the cylinder, directly analogous to Rg for spherical particles.For an infinite length solid cylinder, the relation between R_xc_ and radius r is (Livsey 1987):

3 $$R_{xc}^{2} = \frac{{r^{2} }}{2}$$

### Radius of gyration and the second derivative of the Guinier plot

Small-angle scattering can be used to estimate the radius of gyration of an object (e.g. solid cylinder) because under appropriate assumptions, the Guinier plot is linear over an extended range of q^2^. In this range, the second derivative of the Guinier plot is approximately zero.

In the case of a hierarchical structural ensemble it is possible that the Guinier plot may be linear over multiple regions, each corresponding to a distinct realm of the hierarchical assembly. To test this hypothesis, we calculated the second derivative of the Guinier plot and searched for regions where the second derivative was zero, or close to zero. An obstacle to this calculation is that direct calculation of the second derivative from noisy data—a calculation that leads to a dramatic amplification of noise. Nevertheless, there are computationally efficient approaches to taking these derivatives in the presence of noise. A noise-suppression method using total-variation regularization (Chartrand 2011) was applied here to carry out calculation of the second derivative. Briefly, a derivative is calculated by searching for the function u (the first derivative that minimizes the function F), where *f* is the Guinier plot:

4 $$F(u) = \alpha \int {\left| {u'} \right| + \frac{1}{2}\int {\left| {Au - f} \right|^{2} } }$$

Equatorial traces constructed by merging data from USAXS, VSAXS and SAXS data sets were analyzed according to this method and regions of low second derivative values were identified and utilized to determine the characteristic sizes of the most abundant hierarchical structures in the samples.

## Results

### Cylindrical Guinier analysis on simulated intensities

**Fig. 1:**
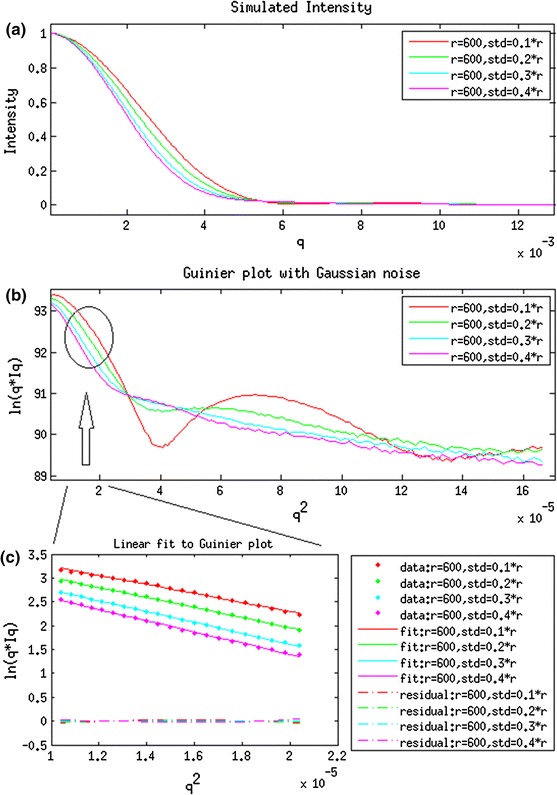
**a** Computationally generated scattering intensities from solid cylinders. **b** Gunier plot of ln[qI(q)] versus q^2^. **c** Linear fit to the low-angle region of Guinier plot

**Fig. 2:**
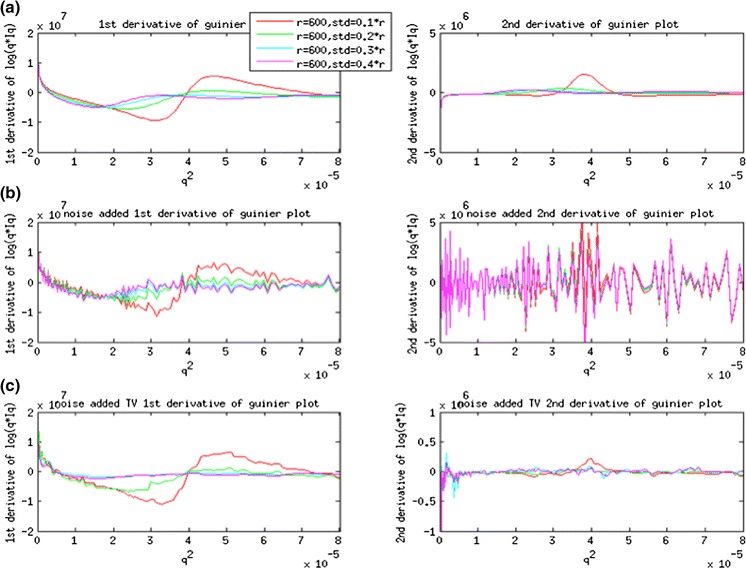
**a** First order (*left*) and second order (*right*) derivative of Guinier plot in noise-free Intensity using numerical differentiation. **b** First order (*left*) and second order (*right*) derivative of Gunier plot with Gaussian noise added to the intensity and using numerical differentiation. **c** First order (*left*) and second order (*right*) derivative of Guinier plot with Gaussian noise added to the intensity and using Total-Variation Regularization method

A set of simulated scattering intensities from cellulose fibrils with different Gaussian distributions of radius was generated using Eq. 1. The cylinder models used to generate the scattering intensities have an average radius of 600 Å and standard deviation of 60, 120, 180 and 240 Å. The Cylindrical Guinier plots of ln(qI) versus q^2^ with and without Gaussian noise are plotted in Fig. 1. The low-angle region is approximately linear for q^2^ < 0.00003 (1/Å) (Fig. 1c). The apparent radius of gyration of cross-section (R_xc_) was calculated based on the slope of the cylindrical Guinier plot. The radius of a solid cylinder is equal to R_xc_/0.707 (Livsey 1987). The Radius of gyration of cross-section (R_xc_) and corresponding radius (r) are tabulated in Table 1. From these simulations, the apparent radius is very close to 600 Å with an error of 2.4 % for distributions of radii with standard deviation of 60 Å. The apparent radius increases with standard deviation because larger radii cylinders contribute more to the intensity than smaller radii cylinders, biasing the resulting intensity towards properties reflecting larger radii. The first and second derivative of the noise-free Guinier plot are in Fig. 2a (1st order on the left and 2nd order on the right). Numerical differentiation of noisy Guinier plots of first and second order are shown in Fig. 2b. Derivatives obtained using Total-Variation Regularization method suppressed the noise and provide the better estimates in Fig. 2c. These results demonstrate the capability of accurate estimation of first and second derivatives in the presence of significant noise levels.

**Table 1:** Calculated R_xc_ and radius of simulated intensities from cylinders

	(r_0_,σ) (Å)	R_xc_ (Å)	r (Å)	Difference between r_0_ and r (%)
1	(600, 60)	434.6	614.6	2.4
2	(600, 120)	458.6	648.4	8.1
3	(600, 180)	475.5	672.4	12.1
4	(600, 240)	483.9	684.3	14.1

### Cylindrical Guinier analysis on USAXS, VSAXS and SAXS intensities

**Fig. 3:**
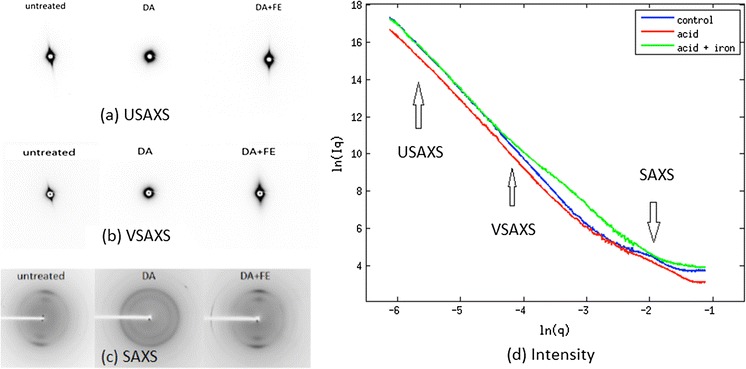
**a** Ultra small-angel X-ray diffraction pattern. **b** Very small-angle X-ray diffraction pattern. **c** Small-angle X-ray diffraction pattern. **d** Combined scattering Intensities of three samples in three length scale

**Fig. 4:**
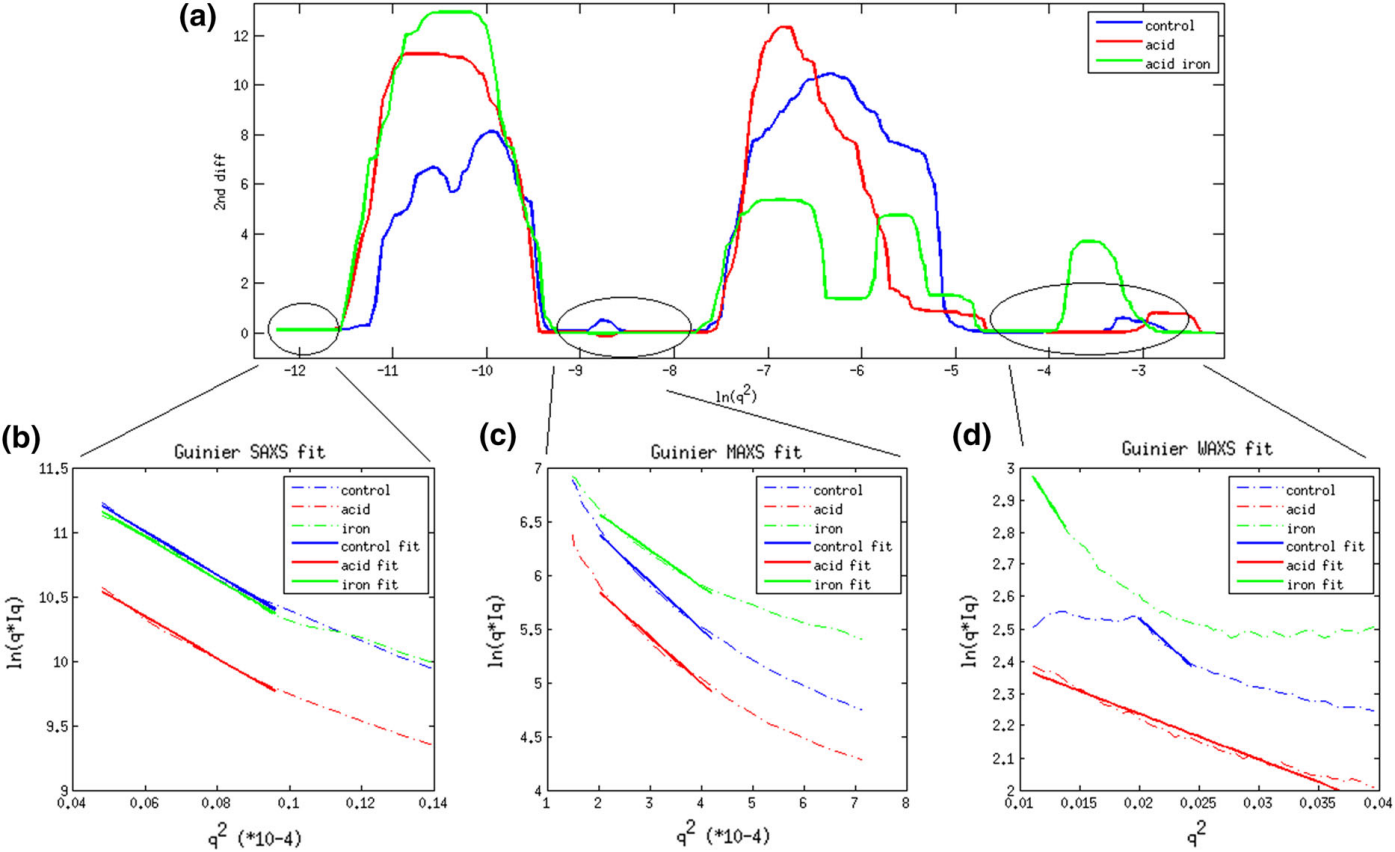
**a** Second order derivatives of Guinier plot on experimental data. **b** Linear fit to Guinier plot in region 1. **c** Linear fit to Gunier plot in region 2. **d** Linear fit to Guinier plot in region 3

USAXS, VSAXS and SAXS patterns are shown in Fig. 3a–c and the equatorial intensities are plotted together in Fig. 3d. The second derivative of the cylindrical Guinier plotted in Fig. 4a approaches zero in three regions. The fitting of Guinier plots from untreated, dilute acid treated and acid plus iron treated maize samples are shown in Fig. 4b–d. The radius of gyration calculated from the linear regions of these plots are provided in Table 2. For USAXS, corresponding to the largest scale structural features in the material, the apparent radii do not significantly change from the control numbers when treated with dilute acid or acid plus iron (Table 2). The VSAXS data indicate a change in apparent size of features in the acid plus iron sample (compared to control or acid treated samples).

**Table 2:** Calculated R_xc_ and radius of scattering intensities among control, acid treated and acid plus iron treated samples

	R_xc_ (Å)	Diameters (Å)
USAXS control	523.5	1481.0
USAXS acid	529.3	1497.2
USAXS iron	536.3	1517.2
VSAXS control	102.2	289.0
VSAXS acid	105.9	299.6
VSAXS iron	92.1	260.6
SAXS control	8.2	23.2
SAXS acid	5.3	15.04
SAXS iron	10.7	30.4

Equatorial intensities in the SAXS patterns are not amenable to so simple an analysis. Equatorial scattering in this regime is informative of the structure and ordering of individual cellulose fibrils. Scattering from untreated material exhibits strong features due to interference effects reflecting side-to-side ordering of cellulose fibrils. Scattering from treated materials does not exhibit interference, suggesting that the treatments lead to disruption of the ordering of fibrils. A naïve calculation of the radii of gyration of treated materials suggests that fibrils in acid-iron samples are about 30 Å in diameter, as expected from earlier studies (Inouye et al. 2014). However, analysis of the acid treated material suggest a much smaller radius, perhaps half that reported previously. It is likely that this discrepancy arises from interference effects due to side-to-side ordering of the fibrils, precluding the estimate of apparent fibrillar radius.

Based on these observations, we conclude that the acid and acid plus iron do not lead to break down of the largest scale structures, the ~0.14 μm bundles. The intermediate-sized structures are, however, highly susceptible to breakdown by acid-iron treatment. This breakdown is consistent with the disappearance of interference effects in the WAXS regime.

Our analyses of scattering data need to be considered in the context of the analyses of simulated data detailed in Figs. 1 and 2. That work indicated that when an ensemble of cylindrical structures was present a Guinier analysis may lead to overestimates of the average diameter. Given the apparent variability of sizes in cellulosic bundles, we presume that our estimate of average size of bundle may overestimate the average size of bundles by as much as 10 %.

## Discussion

Cellulosic biomass is a complex hierarchical structure with well-defined organization on multiple length scales. The substructures observed here have been interpreted in terms of microfibrils, macrofibrils and fibrous bundles, of widely varying dimensions (Himmel 2009). Some of the cellulosic features of different length scales undergo structural changes in response to dilute acid pre-treatments (Nguyen and Tucker 2002; Tucker et al. 2003) whereas others appear recalcitrant to treatment. These structural changes are associated with significant increases in sugar yield on enzymatic digestion, thus represent promising molecular processes for the utilization of bioenergy resources (Inouye et al. 2014). To better understand these structural changes, various imaging techniques such as electron microscopy (SEM and TEM) and X-ray fluorescence microscopy (XFM) (Inouye et al. 2014) have been used to characterize individual structures. Building on that work, we have used X-ray scattering on multiple length scales to characterize different aspect of the structure and changes in cellulosic hierarchical architecture during dilute acid treatment.

We carried out a unified approach, combining data from ultra small angle, very small angle and small angle scattering experiments. The scattering intensities were collected over a very wide range of scattering angles and the data is suggestive of a hierarchy of structural architectures. The Guinier plot [ln(I(q)) vs. q^2^] has been widely used to characterize protein assemblies using solution scattering data where the slope of the linear region of the Guinier plot is proportional to the radius of gyration (Glatter Glatter 1977; Putnam et al. 2007).

For analysis of fibrous structures, an analogous formalism in cylindrical coordinates is required. For this case, a Guinier plot can be constructed of ln(qI(q)) versus q^2^ and the slope of the linear regime is proportional to R_xc_, the radius of gyration of the cross-section of the fibrous structure giving rise to the scattering (Putnam et al. 2007). Here we assert that observation of multiple Guinier regimes reflect multiple length scales where the Guinier conditions are fulfilled and that the presence of multiple regimes reflects a hierarchical architecture with several length scales characteristic of substructures within the material.

We chose to focus on the second derivative of the Guinier plot in order to identify those regimes in which the Guinier conditions are fulfilled. The second derivative of a linear plot is, of course, zero. So regions of the plot in which the second derivative is very low (or zero) should correspond to regions where the Guinier conditions are fulfilled. This raises the challenge of accurate calculation of the second derivative.

The noise in scattering intensities is amplified both by the logarithm and numerical differentiation processes. When applied to experimental data, the signal to noise ratio of the second derivative is very low. Using a total-variation regularization method (Chartrand 2011) we accurately calculated the second derivative of the Guinier plots of our data. This allowed observation of multiple q-ranges in which the second derivative approached zero. The characteristic length scales of the corresponding substructures are then correlated to the slope of the Guinier plot at these positions. The slope of linear fit to Guinier plot was used to calculate R_xc_ of the cross-section of fibrils which was, in turn, used to calculate the diameter of a corresponding cylinder model. This method was applied to both simulated and experimental intensity data, generating estimates for the approximate size of structural features giving rise to the observed intensities.

The first linear region corresponds to cellulose macro fibrils—actually bundles of elementary fibrils—about 0.14 μm in diameter. These structures are resistant to change due to dilute acid as well as acid plus iron pretreatments and results are correlated with Inouye et al. (2014). The acid pretreatment removed non-cellulosic polymeric materials from the surface of microfibrils (Inouye et al. 2014) while potentially depositing sulfate ions on their surface. The presence of iron from Fe_2_(SO_4_)_3_ pretreatment enhanced the electron density contrast on the material, increasing equatorial intensity. Whereas pre-treatments lead to disorientation of microfibrils, larger scale structures including the ~0.14 μ diameter bundles appear to remain oriented within the macroscopic fibrillar context. This disorientation observed by X-ray s may correspond to delamination observed in electron microscopy which also documents broadening of pore and matrix lamella width during pretreatments (Fahlén and Salmén 2005). This behavior can also be observed using SEM (Inouye et al. 2014).

The second linear region demonstrates the presence of an intermediate substructure about 300 Å in diameter. These substructures are apparently bundles of microfibrils that are well ordered within the larger macrofibrils. They exhibit decreased radii after iron-acid pretreatment consistent with partial breakdown of the microfibrils which leads to weaker scattering intensities. These substructures are of a size just below the limit of detectability with SEM. Use of high-pass filtering to process the rare SEM image of a cross-sectional through a ~0.14 μ bundle occasionally resolves structures of the correct size, but not with a frequency to reliably associate them to the ~300 A bundles reported here.

The third regime corresponds to the well characterized elementary microfibrils about 30 Å diameter. The radii of the microfibrils could not be accurately calculated from the small-angle data because of the presence of an interference function due to side-to-side ordering of the fibrils. The dilute acid treatment disrupted the side-to-side organization of the material and diffraction features due to the interference function were essentially eliminated in the acid-iron treated material.

Multiscale analysis of the ultra small, very small and small angle scattering regimes identified unambiguously at least two regions where the cylindrical Guinier plot is approximately linear. This generalization of the classic Guinier analysis supports a broader application of Guinier-type analyses to hierarchical materials with features that may satisfy the Guinier conditions at multiple length scales. We are not aware of a previous observation of this effect.
